# Study of bacterial flora associated with mobile phones of healthcare workers and non-healthcare workers

**Published:** 2017-06

**Authors:** Raghavendra Rao Morubagal, Sowmya Govindanahalli Shivappa, Rashmi Padmanabha Mahale, Sumana Mhadevaiah Neelambike

**Affiliations:** Department of Microbiology, JSS Medical College, JSS University, Shivarathreswara Nager Mysore 570015, Karnataka, India

**Keywords:** Healthcare-associated infections, Mobile phones, *Staphylococcus species*, *Acinetobacter baumannii*

## Abstract

**Background and Objectives:**

Despite improvements in modern diagnosis and therapies, hospital acquired infections remain a leading problem of global health systems. Healthcare workers mobile phones is a reservoir for potential pathogens. Despite the high possibility of being contaminated, mobile phones are rarely clean and are often touched during or after examination of patients and handling of specimens without proper hand washing. The main objective of the present study was to isolate, identify different types of bacteria and their antibiotic sensitivity from mobile phones of healthcare workers and non-health-care workers.

**Materials and Methods:**

Samples were collected aseptically by rolling over the exposed surfaces of the mobile phones inoculated on the agar plates and incubated aerobically. After incubation, plates were examined for growth. Bacteria were identified and antibiotic sensitivity was tested as per standard microbiological procedures.

**Results:**

In this study a total of 175 samples were examined, out of which 125 samples were from healthcare workers (HCWs), 50 samples were from non-healthcare workers (non-HCWs). Among the mobile phones of HCW’s from ICUs, *Acinetobacter baumannii* (36.84%) was the predominant organism isolated followed by methicillin resistant *Staphylococcus aureus* (MRSA) (21.05%). Predominant organism isolated from HCW’s in operation theater theater was MRSA (46.66%). Out of 50 worker’s non-HCWs mobile phones samples cultured, 23 (46.00%) samples yielded growth of six different types of bacteria.

**Conclusion:**

Our study reveals that there is definite colonization of bacteria on mobile phones of the HCWs. It is not only capable of transferring message but also disease-producing microbes. In order to reduce incidence of nosocomial infections, there should be implementation of hand washing practices and regulations around the use of mobile telephones in hospital settings.

## INTRODUCTION

Modern technology has contributed significantly to the field of Medicine, by developing newer techniques in diagnosis, patient-care and treatment, which has significantly increased survival of diseased individual. Modern technology, which is growing at a rapid phase, has also contributed in developing technologies for individual use. This technology includes personal computers, pagers, mobile hand-held devices (wireless tablets, etc.) and mobile phones (1–3). In 1983, in order to improve the communication system, the global system for mobile telecommunication was established in Europe. In India, the first use of mobile phone was in 1995 and today more than 287 million mobile phone users exist, which account for 85% of all the telecommunication users (4, 5). In many countries, mobile phones outnumber landline telephones. Most adult and many children now own mobile phones (6).

Mobile phones increase the speed of communication and contact within healthcare institutions, making healthcare delivery more efficient. Mobile phones dispense laboratory and imaging results, patient data, and photographic images, which are being used by physicians during bedside rounds, in order to engage clinicians, residents, and students. HCWs access pharmaceutical knowledge and literature by mobile phone, which facilitates learning and clinical performance (7, 8). Due to these benefits of mobile phones and computers, their hazard to human health is often overlooked (6). Potential risks of using mobile phones can lead to noise, distractions, loss of concentration, data safety, and disturbance of patient privacy and transfer of micro-organisms possibly leading to nosocomial infections (9). In 2000, World Health Organization (WHO) also described the electromagnetic radiation emitted from phones and base stations as a threat to lives, as it damages the DNA producing sperm cells (10).

The human skin is constantly in contact with micro-organisms and becomes readily colonized by certain microbial species. The adult human is covered with approximately 2m^2^ of skin, with surface area supporting about 10^12^ bacterial cells/person (11). During a phone call, the mobile phone comes into close contact with contaminated human body areas with hands to hands, and hands to other areas like mouth, nose and ears (12), which may result in colonization of potential pathogens present on the human skin, on the mobile phones. In 1997, Aronson et al. first suggested the infection potential of telephones (13).

Despite improvements in modern diagnosis and therapies, healthcare-associated infections (HAIs) remain a leading problem of global health systems. In developing countries, approximately 25% of patients are found to acquire HAIs (14). Hands of HCWs play an important role in the transmission of HAIs. Gowns, gloves, bedside stethoscopes, neckties, bed rails, sheets, telephones, horizontal surfaces, door-knobs, thermometers, nurse’s clothings and personal bags are contaminated by pathogenic bacteria. During daily rounds, hands of HCWs are contaminated with pathogenic bacteria present on these inanimate objects and these bacteria will be transmitted to the patients. HCWs mobile phones provide a reservoir for these potential pathogens. Despite the high possibility of being contaminated, mobile phones are rarely clean and are often touched during or after examination of patients and handling of specimens without proper hand washing. These mobile phones become exogenous sources of infection, for not only the patients but also potential health hazard for workers as well as for family members (15). Further, sharing of cell phones among HCWs and non-HCWs may directly facilitate the spread of potential pathogenic bacteria to the community (16).

The range of micro-organisms, which are present can vary from one person to another, and HCWs may have different hand flora from ordinary members of the public. Bacterial isolates from cell phones of HCWs may vary in numbers and antibiotic sensitivity compared to cell phones of non-medical personnel. Cell phones of HCWs represent a hospital community and non-HCWs represent an environment of community. Hence, the aim of this study was to examine different types of bacteria present in these two categories of personnel’s.

## MATERIALS AND METHODS

### Sample collection.

Samples were collected aseptically with sterile swabs moistened with sterile normal saline and by rolling over the exposed surfaces of the mobile phones. Maximum care was taken to ensure that all the buttons of the keypad, screen, mouthpiece, earpiece, sides and back of the mobiles were properly swabbed since these areas are the most frequent spots, in contact with the fingers.

### Sample inoculation.

After collection, the samples were immediately transported to the laboratory and inoculated on 5% sheep blood agar and Mac-Conkey’s agar and plates were incubated aerobically at 37°C for 24 hours. After incubation, plates were examined for growth and colonial morphology of the isolates. Gram-positive and Gram-negative bacteria were identified as per standard microbiological procedures.

### Antibiotic susceptibility.

Antibiotic sensitivity was tested using the Kirby-Bauer disc diffusion method on Mueller-Hinton agar according to CLSI antibiotic disc susceptibility testing guidelines (17).

The antimicrobial agents tested for Gram-positive cocci were linezolid (30μg), erythromycin (15μg), clindamycin (2μg), ciprofloxacin (5μg), cotrimoxazole (1.25/23.75μg), cefoxitin (30μg) and tetracycline (30μg).

The antimicrobial agents tested for Gram-negative bacilli were pipericillin-tazobactam (100/10 μg), ceftriaxone (30 μg), cefepime (30μg), imipenem (10 μg), cotrimoxazole (1.25/23.75μg), amikacin (30μg), ciprofloxacin (5μg) and ampicillin (10μg).

## RESULTS

In this study, a total of 175 samples were examined, out of which 125 samples were from HCWs and 50 samples were from non-HCWs. From 125 HCW’s mobile phones, 203 bacteria were isolated. Out of which, 90 (43.68%) were staphylococci as the predominant pathogen, followed by 43 (21.18%) *A. baumannii*.

Among HCWs, samples were collected from Doctors, nursing staff, medical students and technicians working in various departments like laboratory, ICUs, Operation Theater and general wards. Fifty mobile samples collected from non-HCWs who were not in contact with patients or not visited hospitals during last one month.

Out of 125 HCWs samples, majority of the samples processed were 29 (23.20%) and 21 (16.80%), from Department of Microbiology and Cardio Thoracic Vascular Surgery operation theater (CTVS), respectively. Among healthcare professionals maximum number of samples processed were 54 (43.20%) and 25 (20%), from staff nurses and doctors, respectively. Area-wise and profession-wise distribution of samples is shown in Table 1.

**Table 1.** Distribution of HCW’s mobile phones samples according to profession and area

**Area**	**Doctors**	**Nurses**	**Technicians**	**Students**	**Area wise distribution of samples**
Department of Microbiology	01	00	12	16	29
Pulmonology ward	01	07	00	02	10
Dialysis	00	08	07	00	15
Respiratory Intensive care unit	00	03	01	00	04
Medicinal Intensive care unit	00	06	00	00	06
Emergency medicinal department	05	11	00	00	16
Operation theater complex	11	01	00	00	12
Genaral ward	03	06	00	00	09
Cardio thoracic vascular surgery operation theater	04	09	04	04	21
Infection control Nurses	00	03	00	00	03
Profession wise distribution of samples	25	54	24	22	125

From 125 HCW’s mobile phones, 203 bacteria were isolated. Out of which, 90 (43.68%) were *Staphylococcus species*, [i.e., MSSA 34 (16.64%), MRSA 31 (15.27%), MSCoNS 09 (4.43%), MRCoNS 12 (5.91%), *S. citreus* 04 (1.97%)] as the predominant pathogen, followed by 43 (21.18%) *A. baumanii*. Different types of bacteria grown from HCWs mobile phone arepresented in Table 2.

**Table 2.** Number and type of bacteria isolated from HCW’s mobile phones

**Isolated organism (n=12)**	**Number of isolated organism (n=203)**	**Percentage (%)**
Methicillin susceptible *Staphylococcus aureus*	34	16.74
Methicillin susceptible coagulase negative *Staphylococci*	09	4.43
Methicillin resistant *Staphylococcus aureus*	31	15.27
Methicillin resistant coagulase negative *Staphylococci*	12	5.91
*Staphylococcus citreus*	04	1.97
Diptheroides	01	0.49
Gram positive spore bearrer	16	7.88
*Pseudomonas aeruginosa*	39	19.21
*Acinetobacter baumannii*	43	21.18
*Klebsiella pneumoniae*	10	4.92
*Citrobacter spp.*	02	0.98
*Escherichia coli*	02	0.98
**Total**	**203**	**100**

Among the mobile phones of HCW’s from ICUs, *A. baumannii* (36.84%) was the predominant organism isolated, followed by MRSA (21.05%). Predominant organism isolated from HCW’s in Operation Theater was MRSA (46.66%).

Among 86 (100%) samples positive for staphylococci, excluding *S. citreus*, 34 (39.53%) were predominantly MSSA. Most of the samples positive for non-fermenters (*A. baumannii* and *P. aeruginosa*) were from HCWs working in ICUs and general wards. Distributation of bacteria isolated from HCWs working in different areas is shown in Table 3.

**Table 3.** Distribution of bacteria isolated from HCW’s mobile phones based on the location.

	MSSA	MSCoNS	MRSA	MRCoNS	*Diptheroides*	*Staphylococcus citreus*	GPB	*K. pneumoniae*	*P. aeruginosa*	*A. baumannii*	*Citrobacter spp.*	*E. coli*	**Number of total isolated organism (n=203)**
Dept of Microbiology	01	01	01	01	01	01	01	01	01	01	01	01	**50**
Wards (pulmonalogy, general medicin, emeregency wards)	00	00	00	00	00	00	00	00	00	00	00	00	**71**
Dialysis Unit	01	01	01	01	01	01	01	01	01	01	01	01	**23**
Operation theater	00	00	00	00	00	00	00	00	00	00	00	00	**15**
Infection control staff Nurses	00	00	00	00	00	00	00	00	00	00	00	00	**06**
ICUS (CTVS, MICU, RICU)	00	00	00	00	00	00	00	00	00	00	00	00	**38**
Total	02	02	02	02	02	02	02	02	02	02	02	02	**203**

MSSA: Methicillin susceptible *Staphylococcus aureus*; MSCoNS: Methicillin susceptible coagulase negative *Staphylococci*; MRSA: Methicillin resistant *Staphylococcus aureus*; MRCoNS: Methicillin resistant coagulase negative staphylococci; GPB: Gram positive spore bearrer

ICUS: Intensive care units; CTVS: Cardio thoracic vascular surgery operation theatre; MICU: Medicinal Intensive care unit; RICU: Respiratory Intensive care unit

Among 25 mobile samples from the Doctors, MRSA (21.95%) was the predominant organism isolated followed by *A. baumannii* (17.07%). Distribution of bacteria from different types of HCW’s is shown in Table 4.

**Table 4.** Distribution of bacteria isolated from HCW’s mobile phones according to the profession

**Isolated micro-organisms (n=203)**	**Doctors (n=25)**	**Nurses (n=54)**	**Technicians (n=24)**	**Student (n=22)**
MSSA (34)	06	13	09	06
MSCoNS (09)	01	03	03	02
MRSA (31)	09	12	05	05
MRCoNS (12)	05	03	01	03
*Staphylococcus citreus* (04)	01	02	01	00
GPB (16)	04	06	03	03
Diptheroides (01)	00	00	00	01
*K. pneumoniae* (10)	02	06	02	00
*P. aeruginosa* (39)	06	21	08	04
*A. baumannii* (43)	07	22	09	05
*Citrobacter spp.* (02)	00	00	02	00
*E. coli* (02)	00	01	00	01
**Total**	41	**89**	**43**	**30**

MSSA: Methicillin susceptible *Staphylococcus aureus*; MSCoNS: Methicillin susceptible coagulase negative *Staphylococci*; MRSA: Methicillin resistant *Staphylococcus aureus*; MRCoNS: Methicillin resistant coagulase negative staphylococci; GPB: Gram positive spore bearrer

In this study, a total 50 non-HCWs mobile phones samples were cultured, out of which, 23 (46.00%) samples yielded growth of six different types of bacteria. Out of which, Gram-positive spore bearer 16 (57.14%) was the predominant organism followed by *Acinetobacter baumanni* (04) (14.28%). Distribution of bacteria from non-HCWs is shown in Table 5.

**Table 5.** Number and type of bacterial agent isolated from mobile phones of Non-HCWs

**Source type**	**Number of samples collected**	**Number of culture positive samples**	**Isolated organisms**	**Number of isolated organisms**
			MSSA	02
			MSCoNS	01
			GPB	16
**Non health care workers**	50	23	*Klebsiella pneumoniae*	03
			*Acinetobacter baumanni*	04
			*Citrobacter spp.*	02
			**Total**	**28**

MSSA: Methicillin susceptible *Staphylococcus aureus*; MSCoNS: Methicillin susceptible coagulase negative *Staphylococci*; GPB: Gram positive spore bearrer

Antibiotic succeptability pattern of staphylococci isolated from HCW’s mobile phones is shown in Table 6. *S. aureus* and coagulase negative staphylococcus (CoNS) were 100% susceptible to linezolid.

**Table 6.** Antibiotic susceptibility pattern of Gram-positive organisms isolated from HCWs

**Antibiotics**	***S. aureus* (n=65)**		***CoNS* (n=21)**	

	**S**	**R**	**S**	**R**
**LZ**	65	00	21	00
**E**	28	37	12	09
**CD**	48	17	15	06
**CIP**	52	13	17	04
**COT**	45	20	16	05
**CX**	34	31	12	09
**TE**	56	09	16	05

LZ: linezolid; E: erythromycin; CD: clindamycin; CIP: ciprofloxacin; COT: cotrimoxazole; CX: cefoxitin; TE: tetracycline; CoNS: Coagulase negative staphylococcus

Among Gram-negative bacteria, 97.67% of *A.baumanii* was susceptible to amikacin, followed by ciprofloxacin (90.69%). 97.43% of *P. aeruginosa* was susceptible to imipenem, followed by amikacin (94.87%). Antibiotic susceptibility pattern of Gram-negative organisms isolated from HCW’s is shown in Table 7.

**Table 7.** Antibiotic susceptibility pattern of Gram-negative organisms isolated from HCW’s

**Antibiotics**	***A. baumannii* (n=43)**		***P. aeruginosa* (n=39)**		***K. pneumoniae* (n=10)**		***E. coli* (n=02)**		***Citrobacter* (n=02)**	

	**S**	**R**	**S**	**R**	**S**	**R**	**S**	**R**	**S**	**R**
**PIT**	26	17	29	10	07	03	01	01	01	01
**CTR**	16	27	15	24	08	02	01	01	02	00
**CPM**	18	25	11	28	09	01	01	01	02	00
**IPM**	31	12	38	01	10	00	02	00	02	00
**COT**	29	14	28	11	08	02	01	01	02	00
**AK**	42	01	37	02	10	00	02	00	02	00
**CIP**	39	04	32	07	09	01	01	01	02	00
**A**	09	34	__	__	01	09	00	02	00	02

PIT: pipericillin-tazobactam; CTR: ceftriaxone; CPM: cefepime; IPM: imipenem; COT: cotrimoxazole; AK: amikacin; CIP: ciprofloxacin; A: ampicillin

Antibiotic susceptibility pattern of staphylococci isolated from non-HCWs showed 100% sensitivity to all antibiotics tested and all the four *A. baumannii* were susceptible to pipericillin-tazobactam, ceftriaxone, cefepime, imipenem, amikacin, cifrofloxacin, and only one isolate was susceptible to ampicillin. All the three *Klebsiella pneumoniae* were susceptible to ceftriaxone, imipenem, amikacin, cifrofloxacin and two isolate were susceptible to pipericillin-tazobactam, cefepime, cotrimoxazole. *Citrobacter spp.* was susceptible to all the antibiotics tested.

## DISCUSSION

The hospital environment plays a very important role in the transmission of micro-organisms causing HAIs. Micro-organisms can be transferred from person to person or from inanimate objects like stethoscopes, bronchoscopes, pens, computer keyboards, mobile phones and fixed telephones to hand and vice versa. In the present study, one such inanimate object mobile phone was studied for microbial colonization.

The mobile phones have become multi-purpose non-medical devices used in the healthcare facility and in the community. It has increasingly become an important means of communication in the community and in the healthcare facility for collecting epidemiological data and monitoring chronic diseases. Mobile phones are used without restriction in healthcare facilities, including specific, susceptible areas like the operation room and ICUs, regardless of their unknown microbial load (18). In a study, it was discovered that cell phonesusually aredirtier than either a toilet seat or the bottom of shoe (4). The constant handling of mobile phones by different users exposes to an array of micro-organisms and thus makes a good carrier for microbes. This is especially so with skin, due to the moisture and optimum temperature of human body especially our palms along with heat generated by mobile phones favors the colonization and multiplication of micro-organisms, so these devices can harbour various potential pathogens and serves as an exogenous source of nosocomial infection among hospitalized patients (6).

In present study, 92.80% of HCWs mobile phones and 57.50% of non-HCWs mobile phones showed microbial growth. Carriage rates of bacterial isolates on cell phones reported by various authors are shown in the Table 8.

**Table 8.** Comparison of carriage rate between studies

**Study**	**Percentage of organisms isolated**	

	**Health care workers**	**Non-health care workers**
Present study	92.80%	57.50 %
Misgana et al. (19)	86.37%	56.06 %
Ulger et al. (20)	94.05%	-
Jaya Lakshmi et al. (21)	91.60%	-
Marwa et al. (22)	92.50%	-
Neha Sharma et al. (23)	94%	80 %

Rate of contamination of mobile phones of HCWs in present study coincides with studies performed-by Marwa et al. (21), Jaya Lakshmi et al. (20), Neha Sharma et al. (22) and Ulger et al. (19). Rate of contamination of mobile phones of non-HCWs reported by Misgana et al. (18) was consistent with the present study. In contrast to the present study, Nehasharma et al. (22) has reported 80% of contamination of mobile phones among non-HCWs. Arora et al. (15) has reported (41%), lower bacterial contamination of mobile phones. The difference in the contamination rate may be due to the variation of the study participants in adherence to infection prevention, the pattern of mobile phone use, mobile phone keeping habits and personal behavior (18). So these finding definitely indicate that HCWs mobile phones were heavily contaminated compared to non-HCWs mobile phones. The reasons for getting a larger number of isolates from HCWs mobile phones may be a consequence of HCWs having direct contact with patients. Non-compliance of hospital standards for infection prevention may also contribute to the finding of high bacterial contamination.

Bacteria known to cause HAIs have varied by clinical settings and have included MRSA, *A. baumannii*, and *Pseudomonas* species (23, 24). Out of 203 bacteria isolated in this study, *Staphylococcal* species (44.33%) were predominant bacteria, grown from HCWs mobile phones. Similar pattern have been observed and reported by Lawani et al. (25). Staphylococcal species especially *S. epidermidis* normally found on skin flora, this might be the reason for their high rate of growth from the mobile phones in the present study. *S. aureus* can cause various illnesses, from minor skin infections to much more serious diseases, which include pneumonia, bacteremia, septicemia etc. MRSA is of particular importance in the medical community, as it has evolved resistance to β-lactam antibiotics (26). Even in the present study, predominant organism isolated from HCW’s in Operation Theater was MRSA (46.66%) and in dialysis unit MRSA (21.73%), followed by MSSA (21.73%).

The second common bacteria isolated from HCWs mobile phones was *A. baumannii.* It is a Gram-negative cocco-bacilli, which are characterized by their truncated rod shape. The organism is ubiquitous, which can be found in the normal skin flora, as well as in soil and bodies of water, amongst others. Multiple drug-resistant strains of *A. baumannii* (MDR) has been arisen, which combined with its ability to persist in hospital environments for extended periods of time, has led to its emergence as a potentially dangerous nosocomial pathogen (27). *A. baumannii* (36.84%) was the predominant organism isolated from mobile phones of HCW’s working in ICUs and from nursing (24.71%) professionals in this study.

One of the alarming signs is that multi-drug resistant organisms like MRSA and *A. baumannii* are isolated from HCWs in critical areas like ICUs, Operation Theater, dialysis units and from doctors and nursing professionals. This could be the reason for high rate of isolation of *A. baumannii* from ventilator-associated pneumonia (VAP) patients in Respiratory Intensive Care Unit (RICUs) and *Staphyloccus* species from post operative wards in our tertiarry care hospital.

In the present study, staphylococci isolated were 100% susceptible to linezolid and 80% were susceptible to ciprofloxacin. Similar findings were also noted by Dardi (28).

*A. baumanii* isolates in our study were susceptible to amikacin (97.67%) and cifrofloxacin (90.69%). *P. aeruginosa* was susceptible to imipenem (97.43%) and amikacin (94.87%). *K. pneumoniae* were susceptible to imipenem (100%), amikacin (100%) and cifrofloxacin (90%). In study performed by Dardi (29), Gram-negative bacilli isolated from mobile phones were 100% susceptible to amikacin, netilmicin, meropenem, ceftazidime, ticarcillin, piperacillin and cefepime.

Out of 50 non-HCWs mobile phone samples examined in this study, 23 (46%) yielded growth. Of these grown isolates, Gram-positive spore bearers (GPSB) were the predominant organisms (n=16, 69.56%). GPSB are non- pathogenic to human beings, may be present on cell phones as contaminants. By excluding growth of GPB, 7 (17.5%) samples yielded the growth of human pathogens. In contrast to the present study, Misgana et al. reported 56.06% (37/66) of growth from non-HCWs mobile phone samples (18). Out of which coagulase negative *Staphylococci* were the predominant organisms.

Several studies also revealed that HCWs do not consider mobile phones to be contaminated items and rarely disinfect their phones (2). Hand washing is the most effective method for the prevention of bacterial transmission. Although there are strict rules on hand hygiene in hospitals, it is not possible to provide de-contamination, disinfection or sterilization of each device used personally. Even though the presence of some items can be restricted in the hospital setting, it is not possible to limit the use of mobile phones by HCWs due to their indispensable benefits. The Centers for Disease Control and Prevention (CDC)’s guidelines for environmental infection control in healthcare facilities recommends periodic disinfection after cleaning instruments and surfaces that often come into contact with the hands, such as computer keyboards and mouse, as defined by the infection control committee (29).

## CONCLUSION

Our study reveals that there is definite colonization of bacteria on the mobile phones, which are very close to the hand of HCWs. Mobile phones are not only capable of transferring messages but also are disease-producing microbes. They may act as a suitable substrate from which the disease may arise, spread and cause havoc, in the form of nosocomial disease. Our study also reveals that colonization of bacteria on the mobile phones of Non-HCW’s is less, compared to HCW’s. These contaminated phones can play a potential role in the spread of drug-resistant bacteria into the community. There should be regulations around the use of mobile telephones in hospital settings due to their potential to contribute to nosocomial infections. Mobile phones of HCWs could be a friend or foe, depending on how it is used during working hours in the hospital.
